# Aspergilosis invasiva con compromiso óseo en lactante de cuatro meses con enfermedad granulomatosa crónica

**DOI:** 10.7705/biomedica.7537

**Published:** 2024-12-23

**Authors:** Héctor Gómez-Tello, Estefany Graciela Mamani-Velásquez, Ana Karen Gómez-Gutiérrez, Carlos Sánchez-Flores, Virginia Lora-Téllez, Sara Espinosa-Padilla, Lizbeth Blancas-Galicia

**Affiliations:** 1 Departamento de Alergias, Hospital para el Niño Poblano, Puebla, México Hospital para el Niño Poblano Hospital para el Niño Poblano Puebla México; 2 Laboratorio de Inmunodeficiencias, Instituto Nacional de Pediatría, Ciudad de México, México Instituto Nacional de Pediatría Instituto Nacional de Pediatría Ciudad de México México; 3 Departamento de Pediatría, Hospital del Niño “Dr. Ovidio Aliaga Uría”, La Paz, Bolivia Hospital del Niño Hospital del Niño La Paz Bolivia; 4 Laboratorio de Micobacteriosis y Micología Médica, Hospital para el Niño Poblano, Puebla, México Hospital para el Niño Poblano Hospital para el Niño Poblano Puebla México

**Keywords:** enfermedad granulomatosa crónica, aspergilosis, aspergilosis pulmonar invasiva, NADPH oxidasa, lactante, enfermedades por inmunodeficiencia primaria., granulomatous disease, chronic, aspergillosis, invasive pulmonary aspergillosis, NADPH oxidase, infant, primary immunodeficiency diseases

## Abstract

La enfermedad granulomatosa crónica es el error innato de la inmunidad que se acompaña con mayor frecuencia de aspergilosis invasiva. En esta enfermedad, la aspergilosis invasiva se presenta en la adolescencia y es rara antes del año de vida. Se presenta el caso de un infante con enfermedad granulomatosa crónica y aspergilosis invasiva.

Se trata de un lactante de sexo masculino de cuatro meses de edad, de madre hipotiroidea y con quien convive en la celda de la cárcel. El infante presentó tumores en la región axilar izquierda y la radiografía de tórax sugirió fracturas costales; fue hospitalizado ante la sospecha de maltrato infantil. En la tomografía de tórax se observó un absceso axilar, osteólisis de costillas, neumonía y nódulos pulmonares; el paciente recibió antibióticos de amplio espectro y fue dado de alta.

A los ocho meses, reingresó por fiebre y extensión del absceso purulento hacia la región escapular izquierda; en la tomografía se observaron imágenes de empeoramiento de la condición. Se aisló *Aspergillus fumigatus* de la secreción del absceso y se diagnosticó aspergilosis invasiva; se inició tratamiento con voriconazol por 28 días. Mediante la prueba de dihidrorrodamina, se diagnosticó enfermedad granulomatosa crónica causada por la variante patógena c.80_83del/Y del gen *CYBB*, portada por la madre (c.80_83del/WT).

A los 12 meses, el paciente reingresó nuevamente por aspergilosis invasiva, resistente al tratamiento, con desenlace fatal.

Este caso excepcional nos enseña que las condiciones ambientales determinan la exposición a los agentes infecciosos en casos de enfermedad granulomatosa crónica y, además, que la aspergilosis invasiva puede presentarse en la infancia y debe tratarse agresivamente.

## Abstract (en)

Chronic granulomatous disease is the inborn error of immunity with the highest frequency of invasive aspergillosis. In this context, invasive aspergillosis is frequent in adolescence, with rare cases before one year of age. We present a case of chronic granulomatous disease and invasive aspergillosis in a four-month-old infant.

The patient was a male infant living in jail with his hypothyroid mother. He presented with a tumor in the left axillary region when he was four months old, and the chest X-ray suggested rib fractures. The patient was hospitalized on suspicion of child abuse. The chest computed tomography scan showed axillary abscess, rib osteolysis, pneumonia, and pulmonary nodules. He was treated with broad-spectrum antibiotics, and then he was discharged.

Four months later, he was readmitted with fever and extension of the purulent abscess to the left scapular region; a computed tomography scan showed worsening images. *Aspergillus fumigatus* was isolated from the abscess pus, leading to an invasive aspergillosis diagnosis. The patient was treated with voriconazole for 28 days, and then he was discharged. The chronic granulomatous disease was diagnosed by the dihydrorhodamine test. The mutated gene causing the inborn error of immunity was *CYBB* with the variant c.80_83del/Y; the mother was the carrier (c.80_83del/WT).

At 12 months of age, the patient was readmitted for invasive aspergillosis, refractory to treatment, and died.

This exceptional case teaches us how environmental conditions determine exposure to infectious agents in chronic granulomatous disease patients. Also, it illustrates that invasive aspergillosis can develope in infants with this pathology and should be treated aggressively.

La enfermedad granulomatosa crónica es un error innato de la inmunidad causante de un defecto de la fagocitosis. Los pacientes tienen una alteración en la producción de especies reactivas de oxígeno por alteración de la enzima NADPH oxidasa.

El cuadro clínico se caracteriza por inflamación e infecciones, en diferentes órganos, causadas por hongos y bacterias. Entre los agentes fúngicos, *Aspergillus* spp. es el más frecuente ^1^. La incidencia anual de la aspergilosis invasiva en pacientes con enfermedad granulomatosa crónica en los Estados Unidos es del 6,5 % ^2^. Esta infección comúnmente afecta los pulmones, pero puede diseminarse a los huesos ^3^ y se asocia con una mortalidad del 20 al 30 % ^4^. En promedio, los pacientes con enfermedad granulomatosa crónica tienen su primer episodio de aspergilosis invasiva a los 10 años ^4^^,^^5^, por lo que son sumamente raros los casos que se manifiesten antes del primer año.

En este reporte, se presenta el caso de un paciente de cuatro meses con aspergilosis invasiva en las costillas, como primera manifestación de enfermedad granulomatosa crónica.

## Caso clínico

**Figura 1.**
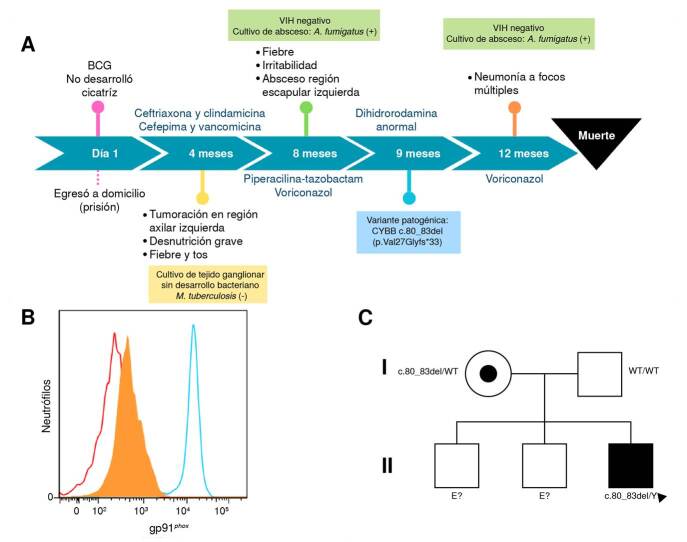
A) Línea del tiempo de la evolución del paciente. B) Histograma de la expresión de gp91 phox en neutrófilos: se representa el isotipo (rojo) y la expresión del paciente (naranja) y del control sano (azul). C) Árbol genealógico del paciente (cuadro negro).

Se trata de un paciente de sexo masculino de cuatro meses de edad, originario del centro de México y con dos hermanos hombres sanos. Durante el primer trimestre de gestación, su madre hipotiroidea consumió drogas. No obstante, el infante nació con peso y talla adecuados, fue alimentado con leche materna y recibió la vacuna del bacilo de Calmette-Guérin (BCG), sin desarrollo de cicatriz (figura 1 A). La madre del paciente era prisionera en una cárcel y, junto con el bebé, compartían una celda insalubre con dos personas más.

A los cuatro meses, el paciente fue valorado médicamente por un tumor doloroso en la región axilar izquierda. En la radiografía de tórax se observaron imágenes sugestivas de fracturas costales; se sospechó de maltrato infantil por parte de la madre, y el infante fue hospitalizado en un hospital pediátrico. Se determinó el peso (4.190 g) y la talla (58 cm) -por debajo del percentil tres-, saturación de oxígeno del 70 %, fiebre, tos, aumento de volumen en la región axilar izquierda, además de dolor, rubor y calor.

En el cuadro hemático se encontró: hemoglobina de 8,8 g/dl (11,0 - 12,6), 29,3 × 109 leucocitos/L (6,0 - 17,5), 18,4 × 109 neutrófilos/L (1,0 - 8,5), 7,0 × 109 linfocitos/L (4,0 - 13,5), 3,5 × 109 monocitos/L, 459 × 109 plaquetas/L (150 - 350) y proteína C reactiva igual a 16 mg/L (< 3,0).

**Figura 2.**
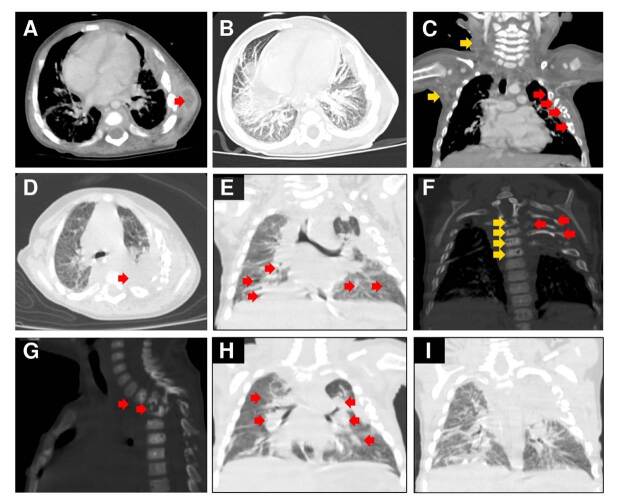
Tomografía computarizada de tórax. **A)** Absceso en la región axilar izquierda (flecha). **B)** Nódulos y micronódulos pulmonares bilaterales. **C)** Lesiones líticas en las costillas 3 a 6 (flechas rojas), conglomerado de ganglios cervicales y axilares (flechas amarillas). **D)** Neumonía apical izquierda (flecha). **E)** Bronquiectasias (flechas). **F)** Vista frontal de las lesiones líticas en las costillas 2 a 7 (flechas rojas) y en las vértebras dorsales 2-7 (flechas amarillas). **G)** Vista lateral de las lesiones líticas en las vértebras dorsales 2 a 7 (flechas). **H)** Neumonía multifocal (flechas); **I)** Fibrosis y retracción bilateral del parénquima pulmonar.

En la primera tomografía toracoabdominal se observaron imágenes de un absceso en la región axilar izquierda, lesiones líticas en las costillas 3 a 6, neumonía apical izquierda, nódulos pulmonares en ambos pulmones y ganglios linfáticos cervicales y mediastinales incrementados de tamaño (figura 2, A-C). En la biopsia del absceso axilar izquierdo se reportó miositis y paniculitis supurativa. Solo se hizo cultivo para bacterias del líquido broncoalveolar, el cual fue negativo, y la PCR para el complejo *Mycobacterium tuberculosis* fue negativa. Después de 41 días de hospitalización y de haber recibido dos esquemas de antimicrobianos -ceftriaxona-clindamicina y cefepima-vancomicina-, el paciente fue dado de alta.

Dos meses después, a los ocho meses de edad, reingresó al hospital por fiebre, irritabilidad y un absceso supurante en la escápula izquierda. En el cuadro flemático se encontró: hemoglobina de 10,8 g/dl (10,5 -12), 21,2 x 10^9^ leucocitos/L (6 -17), 12,2 x 10^9^ neutrófilos/L (1,5 - 8,5), 7,5 x 10^9^ linfocitos/L (4 -10,5), 1,2 x 10^9^ monocitos/L (600), y 583 x 10^9^ plaquetas/L (150 - 350); la prueba para la detección sérica de HIV fue negativa.

En la tomografía de tórax se observó una consolidación apical izquierda, bronquiectasias, lesiones líticas en las costillas 2 a 7 y en las vértebras dorsales 2 a 7, además de una colección de líquido multiloculado (figura 2, D-G); en el ultrasonido se encontró una fístula asociada con el absceso escapular. El paciente recibió piperacilina-tazobactam, que se sustituyó después por voriconazol al detectarse *Aspergillus fumigatus* en el cultivo de la muestra de secreción del absceso.

Dada la recurrencia y la gravedad de la infección, se sospechó un error innato de la inmunidad. La prueba de dihidrorrodamina resultó sin producción de especies reactivas de oxígeno y la expresión de gp91^*phox*^ en los neutrófilos fue nula, estableciéndose así el diagnóstico de enfermedad granulomatosa crónica ligada al cromosoma X (figura 1B). La variante patógena detectada por la secuenciación de nueva generación fue c.80_83del/Y (p.Val27Glyfs*33) en *CYBB*. La madre resultó portadora de la variante (c.80_83del/WT). No fue posible estudiar genéticamente a sus dos hermanos mayores, de sexo masculino, y aparentemente sanos (figura 1C).

El paciente fue dado de alta después de 65 días de hospitalización y tratamiento con voriconazol por 28 días. Se inició profilaxis antibiótica diaria con trimetoprim-sulfametoxazol y profilaxis antifúngica con fluconazol dos veces a la semana.

Dos meses después, al año de edad, el infante reingresó debido a una neumonía multifocal (figura 2, H-I), por la cual requirió asistencia respiratoria mecánica. Se detectó el antígeno galactomanano en el suero y *A. fumigatus* en el cultivo del líquido del lavado broncoalveolar, por lo que se inició nuevamente tratamiento con voriconazol.

El paciente sufrió una falla orgánica múltiple y falleció un mes después de su ingreso.

### Consideraciones éticas

Los autores declaran haber seguido los protocolos de su centro de trabajo sobre la publicación de datos de pacientes y la firma del consentimiento informado.

## Discusión

Se presenta el caso de un paciente de cuatro meses con aspergilosis invasiva en las costillas, como primera manifestación de enfermedad granulomatosa crónica. En los estudios internacionales, se ha documentado el inicio de la aspergilosis invasiva a los 10 años ^4^; sin embargo, este paciente la padeció antes de los cuatro meses. Hasta la fecha, en la literatura científica consultada, solo se han reportado nueve casos (incluido este paciente) de aspergilosis invasiva con inicio antes del año de edad como primera manifestación de enfermedad granulomatosa crónica (cuadro 1).

**Cuadro 1:** Lactantes con aspergilosis invasiva y enfermedad granulomatosa crónica

Código del paciente	Sexo	Gen	**Edad de inicio de síntomas**	Hallazgos clínicos de aspergilosis	**Método de detección de *Aspergillus* spp.**	Factor de riesgo para aspergilosis	Estudios de imágenes diagnósticas	Tratamiento antifúngico	Resultado
País (año)									
Caso reportado México (2024)	M	*CYBB*	4 meses	Fiebre, tos, absceso axilar izquierdo	*A. fumigatus*: muestra de lavado broncoalveolar y secreción de absceso Detección positiva de galactomanano	Ambiente insalubre, hacinamiento	Tomografía computarizada: lesiones líticas en las costillas, neumonía apical izquierda, nódulos pulmonares, ganglios linfáticos cervicales y mediastínicos agrandados	Voriconazol	Fallecido
P1 Japón(2014)^6^	F	*NCF2*	22 días	Dificultad respiratoria, tos, fiebre y rinorrea	*A. fumigatus*: muestra de tejido pulmonar Detección positiva de galactomanano y (3-D glucano	Edificio en demolición	Tomografía computarizada: nódulos bilaterales, difusos y dispersos; consolidaciones y atelectasias en diferentes áreas pulmonares	Voriconazol, micafungina, anfotericina B liposómica	Vivo
P2 Korea (2020) ^7^	M	*CYBB*	24 días	Fiebre y dificultad en la alimentación	Hifas de aspergillus: histología de tejido pulmonar	NE	Tomografía computarizada: masa en el lóbulo inferior derecho y nodulo en leí óbulo medio derecho; derrame pleural	NE	Vivo
P3 Estados Unidos (2002) ^8^	M	*CYBB*	15 días	Fiebre	*Aspergillus* spp.: muestra de tejido pulmonar	NE	Tomografía computarizada: nódulos pulmonares	NE	Vivo
P4 Singapur (1994) ^9^	F	*NCF1*	3 meses	Tos, disnea, falla de medro	*Aspergillus* spp.: muestra de tejido pulmonar	NE	Radiografía de tórax: neumonía bilateral	Anfotericina B liposómica (ocho semanas)	Vivo
P5 Estados Unidos (2015) ^10^	M	*CYBB*	21 días	Fiebre, taquipnea, irritabilidad	*Aspergillus* spp.: muestra de tejido pulmonar	NE	Tomografía computarizada: nódulos y masas pulmonares bilaterales	Voriconazol	Vivo
P6 Estados Unidos (2007) ^11^	F	*CYBA*	21 días	Fiebre, irritabilidad, disminución en la ingesta oral	Hifas de Aspergillus: histología de tejido pulmonar	NE	Tomografía computarizada: múltiples masas pulmonares	NE	NE
P7 Francia (1995) ^12^	M	*CYBB*	27 días	Fiebre, dificultad respiratoria	*A. fumigatus*: muestras de lavado broncoalveolar y ganglios linfáticos mediastinales	NE	Tomografía computarizada: múltiples nódulos pulmonares intraparenquimatosos bilaterales	Anfotericina B liposómica (12 meses)	NE
P8 China (2020) ^13^	M	*NFC2*	1 mes	Fiebre, tos	*A. fumigatus*: muestras de lavado broncoalveolar	NE	Tomografía computarizada: nódulos y masas pulmonares	Voriconazol	Vivo
P9 Alemania (2002) ^14^	F	*NE*	1 mes	Dificultad respiratoria	A*spergillus* spp.: muestra de lavado broncoalveolar	NE	Tomografía computarizada: Nódulos y masas pulmonares	Anfotericina B liposómica	Vivo

NE: no especifica

*Aspergillus* spp. es un hongo saprofito, inocuo, que resiste altas temperaturas, por lo que la inhalación de las conidiosporas distribuidas en el ambiente es habitual. Las áreas húmedas, con poca higiene y mantenimiento, como los edificios en construcción, tienen una concentración elevada de conidiosporas y los pacientes con enfermedad granulomatosa crónica pueden infectarse ^15^. El paciente aquí reportado estuvo expuesto a un área insalubre (celda de prisión) después de su nacimiento, siendo este el factor de riesgo para el inicio temprano de la infección.

La presentación clínica de la aspergilosis invasiva puede ser variable. Inicialmente, hasta el 33 % de los pacientes pueden ser asintomáticos ^1^. Se ha reportado fiebre en el 20 al 61 % de los casos, y puede presentarse leucocitosis y elevación moderada de la velocidad de sedimentación globular o no presentarse ^1^^,^^3^. En ocho de los nueve casos hasta ahora reportados, en quienes la aspergilosis invasiva se inició antes del año de edad, se presentó fiebre; además hubo otros síntomas inespecíficos, como irritabilidad, tos, dificultad respiratoria y alimentaria, y falla de medro (cuadro 1).

El presente paciente tuvo, como primera manifestación, inflamación en la región axilar izquierda sin fiebre. En su primera radiografía de tórax se observaron cambios que sugerían fracturas costales que, en su contexto social, hizo sospechar maltrato infantil.

En un metaanálisis de 116 pacientes con enfermedad granulomatosa crónica y aspergilosis invasiva se describió que el principal órgano afectado fue el pulmón (71 %), seguido del sistema óseo (40 %) ^3^. En los nueve pacientes reportados con aspergilosis invasiva iniciada antes del año, el único órgano afectado fue el pulmón (cuadro 1). En el presente caso, la infección se detectó por el daño óseo, pero, tras los estudios diagnósticos, se encontró que la aspergilosis invasiva también comprometía pulmón, músculo y piel.

El aislamiento del agente infeccioso en los casos de enfermedad granulomatosa crónica es importante para diferenciar *Aspergillus* spp. de otros hongos filamentosos. El lavado broncoalveolar, la aspiración con aguja fina y la biopsia percutánea transtorácica o toracoscópica video-asistida, se encuentran entre las técnicas más utilizadas para el diagnóstico basado en el cultivo ^4^. Se recomienda la biopsia de pulmón, ya que aumenta la tasa de detección de agentes patógenos del 30 al 50 % en los pacientes con enfermedad granulomatosa crónica ^4^.

Existen otras pruebas de diagnóstico, como la detección de galactomanano en los distintos fluidos corporales ^4^; sin embargo, solo resulta positiva en una cuarta parte de los pacientes infectados. Los títulos de galactomanano son proporcionales a la carga fúngica del tejido estudiado ^16^. En los nueve pacientes reportados con inicio del compromiso antes del año (cuadro 1), se utilizaron diferentes técnicas para aislar el agente, como el cultivo (mediante biopsia pulmonar y lavado broncoalveolar) y la detección de galactomanano. En el paciente de este reporte, se aisló *A. fumigatus* mediante el cultivo de una muestra de la secreción del absceso en la región axilar izquierda y detección de galactomanano (positivo), lo cual denota la alta carga fúngica del paciente.

Los estudios de imágenes diagnósticas ayudan a localizar y evaluar el alcance de la infección. En la radiografía de tórax se suelen observar cambios inespecíficos, por lo que la tomografía computarizada se ha convertido en la herramienta principal ante la sospecha de aspergilosis invasiva ^4^. Entre los hallazgos reportados en los estudios pediátricos, se destacan las opacidades nodulares (59 - 100 %), la consolidación (21 - 63 %), la cavitación (0 - 43 %) y el signo de la media luna de aire (0 - 21 %) ^17^. La extensión local del parénquima pulmonar a las estructuras adyacentes y la osteomielitis de la caja torácica son hallazgos particularmente asociados con la aspergilosis invasiva en la enfermedad granulomatosa crónica ^4^.

En los nueve pacientes documentados en el cuadro 1, se reportaron nódulos pulmonares en ocho, observados en la tomografía computarizada. Por el contrario, no se reportó afección de los tejidos adyacentes al pulmón. En el paciente aquí presentado, la radiografía inicial de tórax sugería fracturas costales; sin embargo, en la tomografía computarizada de tórax se observaron nódulos pulmonares, lesiones líticas en las costillas, neumonía apical izquierda y ganglios linfáticos cervicales y mediastinales agrandados, que apoyaron el diagnóstico de aspergilosis invasiva. No obstante, fue hasta la segunda hospitalización que se hizo el diagnóstico.

Las guías prácticas para el tratamiento de la aspergilosis ^18^^,^^19^ mencionan al voriconazol como la primera opción de tratamiento contra la aspergilosis invasiva; los fármacos alternativos son anfotericina B liposómica y desoxicolato, posaconazol, isavuconazol, caspofungina y micafungina ^18^^,^^19^. El voriconazol en menores de dos años debe administrarse con seguimiento para ajustar la dosis o, incluso, considerar dosificaciones más frecuentes ^20^. Se debe contemplar el cambio de la terapia intravenosa a la oral en pacientes clínicamente estables, con absorción entérica confiable. No existe evidencia sólida para determinar la duración óptima del tratamiento; puede ser hasta de 12 semanas, pero se sugiere individualizarlo en función de la mejoría clínica y la inmunosupresión subyacente ^18^^,^^19^.

En los nueve pacientes con enfermedad granulomatosa crónica y aspergilosis invasiva de inicio antes del año (cuadro 1), los tratamientos fueron voriconazol en dos pacientes, anfotericina B liposómica en otros dos, y voriconazol, micafungina y anfotericina B liposómica consecutivamente en un paciente. Se reportó una duración de 8 y 12 semanas en dos pacientes; en el resto no se especificó.

El paciente del presente reporte fue hospitalizado en tres ocasiones; en la primera, recibió antibióticos de amplio espectro, y en la segunda, recibió nuevamente antibióticos; después del aislamiento de *A. fumigatus*, se administró voriconazol por 28 días y, en la tercera hospitalización, también se inició voriconazol, hasta su deceso. Considerando el padecimiento de base, el paciente tenía un gran riesgo de morbimortalidad, por lo que el tratamiento antifúngico fue tardío y de corta duración, con relación a lo recomendado por guías de práctica clínica. La profilaxis con antifúngicos se ha descrito con dosis diarias ^18^^,^^19^^,^^21^; sin embargo, en este caso, el paciente recibió fluconazol solo dos veces por semana, lo cual pudo contribuir a la reinfección fúngica.
